# UCA1 executes an oncogenic role in pancreatic cancer by regulating miR-582-5p/BRCC3

**DOI:** 10.3389/fonc.2023.1133200

**Published:** 2023-07-25

**Authors:** Xiaole Hu, Jiahao Wu, Jianwei Xu

**Affiliations:** ^1^ Department of First Operating Room, Qilu Hospital of Shandong University, Jinan, China; ^2^ Department of Pancreatic Surgery, General Surgery, Qilu Hospital, Shandong University, Jinan, China

**Keywords:** pancreatic cancer, lncRNA, miRNA, prognosis, ceRNA

## Abstract

**Background:**

As a fatal disease, the mechanism of pancreatic cancer is unclear. Urothelial carcinoma antigen 1(UCA1), a long noncoding RNA (lncRNA) that was first reported in bladder cancer, acts as an oncogene. However, the regulatory role and mechanism of UCA1 in pancreatic cancer remain unknown. This study aims to investigate the expression level and prognostic value of UCA1 in pancreatic cancer tissues, the effects and mechanism of UCA1 in regulating cell proliferation, apoptosis and metastasis.

**Methods:**

UCA1 expression levels in tissues were detected by in situ hybridization (ISH) and the prognostic value was evaluated by univariate and multivariate survival analysis. For *in vitro* experiments, proliferation was evaluated by a cell count kit assay, Edu experiments, and a clone formation assay. Apoptosis was evaluated by fluorescence-activated cell sorting flow-cytometry. Cell migration and invasion capacities were detected by wound healing and transwell assays. Western blots were performed to detect apoptotic associated molecules and epithelial-mesenchymal transition (EMT) markers. For the *in vivo* experiment, subcutaneous transplantation models of pancreatic cancer in nude mice were established to observe the tumor growth. The regulatory mechanism of UCA1 was explored by proteomics, bioinformatic analysis, luciferase reporter assays, and rescue experiments.

**Results:**

ISH staining revealed that UCA1 levels between cancer tissues (n=94) and tumor-adjacent tissues (n=73) did not show significant differences. Survival analysis indicated that high expression of UCA1 was an unfavorable prognosis factor for pancreatic cancer. Downregulation of UCA1 by siRNA significantly inhibited cell proliferation, decreased the capacities of cell migration and invasion, induced cell apoptosis, and inhibited EMT. Furthermore, we demonstrated that UCA1 positively regulated the expression of BRCC3 by inhibiting miR-582-5p. Rescue experiments indicated that either inhibiting the expression of miR-582-5p or enhancing expression of BRCC3 could partly attenuate the antitumor effects of downregulation of UCA1.

**Conclusion:**

UCA1 acted as an oncogene in pancreatic cancer by partly regulating miR-582-5p/BRCC3, which could be a new therapeutic target for pancreatic cancer.

## 1 Introduction

Pancreatic cancer is a fatal disease with a 5-year survival rate of less than 10% (1). Besides the classic chemotherapy and radiotherapy, several novel therapeutic regimens have been developed in an attempt to improve the prognosis of pancreatic cancer, such as immunotherapy and targeted therapy. However, the outcomes of these treatments have not met expectations (2). The complex mechanisms of tumorigenesis and development may partly account for the poor prognosis. Well understanding of the mechanism may be benefit to improve the therapeutic effects and then the prognosis.

Long noncoding RNA (lncRNA) is involved in the regulation of multiple biological processes in many cancers, such as tumor tumorigenesis, development, metabolism, proliferation, apoptosis, metastasis, resistance to chemotherapy, and immune escape (3). The crucial role of lncRNA has also been investigated in pancreatic cancer. Some lncRNAs dysregulated in pancreatic cancer, such as HOTAIR, XIST, GAS5, NEAT1, which promoted or inhibited tumor progression through sponging miRNA or binding to transcription factors (4).

Urothelial carcinoma antigen 1 (UCA1) was first discovered in bladder cancer (5), which promoted tumor progression in bladder and prostate cancer (6).With further research, the role of UCA1 in gastrointestinal cancers has also been reported. The expression of UCA1 was increased in tissues or cell lines of gastrointestinal cancers, such as gastric cancer, colorectal cancer, hepatocellular carcinoma, which promoted cell proliferation, metastasis, and drug resistance by interacting with miRNAs (7, 8). Circulating UCA1 levels showed potential value in diagnosis of early-stage colorectal cancer (9) and in predicting the prognosis of patients with gastric cancer (10). Similarly, UCA1 enhanced tumor growth and metastasis in pancreatic cancer by regulating miR-590-3p/Kras (11) and miR-96/FOXO3 (12). Recently, Sun et al. reviewed the functions and pointed out the importance of UCA1 in pancreatic cancer, who showed that UCA1 contributed to carcinogenesis, angiogenesis, and drug resistance via several signal pathways (13). Despite this, few details about UCA1 in pancreatic cancer are known, the regulatory role and mechanism of UCA1 are still unclear. The aim of this study is to explore and reveal the regulatory effect and mechanism of UCA1 in pancreatic cancer through *in vitro* and *in vivo* experiments.

## 2 Materials and methods

### 2.1 Ethics statement

This study was approved by the Medical Ethics Committee of Qilu Hospital of Shandong University (No. KYLL-2019(KS)-139) and the Institutional Animal Care and Use Committee (DWLL-2020-055).

### 2.2 Tissue collection and in situ hybridization

Formalin-fixed, paraffin-embedded pancreatic cancer tissues (n=94) and adjacent normal tissues (n=73) were collected. A locked nucleic acid probe (Exiqon, Vedbaek, Denmark) was used to detect UCA1 expression levels in tissues. ISH was performed as the previous report (14).

The slides were scored by two independent pathologists at our institute according to the intensity of staining and the number of positive cells. Scoring for staining intensity was as follows: none (0 point), weak staining (1 points), intermediate staining (2 points), and strong staining (3 points). Scoring for the percentage of positive cells was as follows: absent (0 point), 1–24% positive cells (1 point), 25–49% (2 points), 50–74% (3 points), and 75–100% (4 points). The final score was calculated by multiplication of the above two scores. The expression of UCA1 was considered low if the final score was less than 2 points and high if the final score was 2 or more points.

### 2.3 Cell lines and culture

Pancreatic cancer cell lines (AsPc-1 and Panc-1) and HEK-293 cell line were a gift from Gang Yang from Peking Union Medical College, which were cultured in a humidified incubator with 5% CO2 at 37°C in RPMI-1640 medium or Dulbecco’s modified Eagle’s medium (DMEM, Hyclone, Thermo Fisher Scientific Inc., Waltham, MA) containing 10% fetal bovine serum (FBS; Hyclone).

### 2.4 Cell transfection

UCA1 were downregulated by transfection with siRNA (named si-UCA1-1 and si-UCA1-2). MiR-582-5p were downregulated and upregulated by transfecting inhibitors and mimics, respectively. Both UCA1 and BRCA1-BRCA2-containing complex subunit 3 (BRCC3) were upregulated by transfecting the overexpression plasmids that the cDNAs were inserted into eukaryotic expression vector pcDNA3.1 plasmid. All the reagents were purchased from RiboBio (Guangzhou, China) and transfected using Lipofectamine 3000 (Invitrogen). The siRNA sequences are listed in **Additional File 1**.

### 2.5 Cell growth assay

Cell growth was detected using a Cell Counting Kit-8 reagent (CCK-8, Dojindo, Tokyo, Japan) according to the manufacturer’s protocol. Pancreatic cancer cells were transfected in six-well plates (5×10^5^ cells/well) for 24 h, then trypsinized and reseeded in 96-well plates (1000 cells/well). CCK-8 (10 μl/well) was added at 0, 24, 48 and 72 hours, respectively. After an additional incubation of 2.5 h at 37°C. optical density (OD) was measured at the wavelength of 450 nm (OD450) using a microplate reader (Wellscan MK3, Thermo/Labsystems, Finland).

### 2.6 5-Ethynyl-2’-deoxyuridine experiments to detect cell proliferation and viability

Pancreatic cancer cells were incubated with EdU (50μM) for 2 hours and then fixed with 4% paraformaldehyde for 30 min. After washing three times with phosphate buffered saline (PBS), cells were permeabilized with Triton X-100 for 20 min. The cells were then incubated away from light with 500μL Click Additive Solution for 30 min. After washing with PBS, cells were stained with Hoechst 33342 for 15 min. Pictures were captured with a fluorescence microscope.

### 2.7 Colony-formation assay

Pancreatic cancer cells were seeded in a six-well plate and incubated for two weeks. After washing with PBS, the cells were fixed with 4% paraformaldehyde, then stained and photographed.

### 2.8 Wound healing assay

Pancreatic cancer cells were seeded in a six-well plate and incubated for 12 hours. Then the cells were scraped for a 250μm scratch with a pipette tip, and the cell fragments were cleaned with PBS. Cells were photographed immediately with a microscopy and 24, 48 hours later.

### 2.9 Transwell assay

Pancreatic cancer cells were seeded in medium without FBS in the upper chamber of a Matrigel transwell system (polycarbonate membrane, diameter 6.5 mm, pore size 8 μm,; Corning Costar, USA). Media with 10% FBS was added into the bottom chamber. After 24˜48 hours of incubation, invaded cells were fixed with 4% paraformaldehyde and stained with hematoxylin and eosin (Servicebio, Wuhan, China). Cell numbers were counted under a microscope in five random visual fields.

### 2.10 Apoptosis assay

Pancreatic cancer cells were transfected in six-well plates. 48 hours later, cells were harvested and resuspended in a binding buffer. The cells were then stained with annexin V-FITC and PI (Beyotime, China) according to the manufacturer’s instructions and analyzed using flow cytometry (FACScan; BD Biosciences, USA).

### 2.11 Luciferase reporter assay

Wildtype or mutant miR-582-5p binding site sequences in BRCC3 were synthesized by Invitrogen and cloned into pmirGLO vectors 3’ of the firefly luciferase gene. Vectors and miR-582-5p mimics or control were cotransfected into HEK-293 cells using Lipofectamine 3000. After 48hours, luciferase activities were evaluated using the Dual-Luciferase Reporter Assay System (Promega) according to the manufacturer’s guidelines.

### 2.12 qRT-PCR

Total cellular RNAs were extracted using TRIzol (Invitrogen) according to the manufacturer’s introduction. Total RNAs were reverse transcribed using the reverse transcription kit (Promega, Madison, WI) according to the manufacturer’s guidelines. qRT-PCR was performed using the SYBR Green. GAPDH and U6 were used as internal controls to detect mRNA and miRNA, respectively. Gene expression levels were calculated using the 2^-ΔΔCT^ methods. The sequences of primers are listed in **Additional File 1**.

### 2.13 Western blot

Total proteins were extracted from cells using a RIPA buffer (Applygen, Beijing, China), then separated using sodium dodecyl sulphate-polyacrylamide gel electrophoresis and transferred to polyvinylidene difluoride membranes (Millipore, Billerica, MA, USA). After blocking in 5% skim milk at room temperature for 1 hour, the membranes were incubated with primary antibodies overnight at 4°C. The membranes were then probed with horseradish peroxidase–conjugated secondary antibodies at room temperature for 1 hour. Bands were visualized using an echochemiluminescence (ECL) detection system (Amersham Biosciences, Foster City, CA, USA). Band intensities were quantified using Image-Pro Plus 6.0 software (Media Cybernetics, USA).

Details about the primary antibodies are as follows: Bax (1:2000, Biorbyt, orb4655), Bcl-2 (1:1000, Biorbyt, orb135113), cytochrome c (1:1000, Biorbyt, orb539087), N-cadherin (1:500, Biorbyt, orb238690), E-cadherin (1:1000, Biorbyt, orb213706), Vimentin (1:2000, Biorbyt, orb158714), Snail (1:500, Biorbyt, orb180479), Slug (1:1000, Biorbyt, orb33656), Twist1 (1:1000, Biorbyt, orb542191), ZEB1 (1:1000, Biorbyt, orb12781), GAPDH (1:1000, Biorbyt, orb38656), BRCC3 (1:1000, MyBioSource, MBS607029).

### 2.14 Animal experiments

Panc-1 cells were injected subcutaneously into the right back of 6-week-old female BALB/c mice (1×10^7^ cells in 200 μl PBS per mouse). Each group contained five mice. The tumor size was measured twice a week using caliper measurement of two perpendicular diameters of the implants. Tumor volume (mm^3^) was calculated according to the formula: volume (mm^3^) = 1/2 × length × width^2^. The tumor-bearing mice were euthanized on the 30^th^ day.

The xenografted tumors were harvested. Terminal-deoxynucleotidyl transferase mediated nick end labeling (Tunnel) assay was performed to detect apoptosis according to the introduction (Beyotime, China). Immunohistochemistry (IHC) was performed to detect Ki-67 (Abcam, ab15580). IHC was carried out as the previous report (15).

### 2.15 Proteomic analysis

#### 2.15.1 Protein extraction and trypsin digestion

Proteins were extracted from pancreatic cancer cells and the concentration was determined with BCA kit (Beyotime) according to the manufacturer’s instructions. Then, the protein solution was reduced with 5 mM dithiothreitol for 30 min at 56°C and alkylated with 11 mM iodoacetamide for 15 min at room temperature in darkness. The protein sample was then diluted by adding 100 mM triethylammonium bicarbonate (TEAB, Sigma) to a urea concentration less than 2M. Finally, trypsin was added at 1:50 trypsin-to-protein mass ratio for the first digestion overnight and 1:100 trypsin-to-protein mass ratio for a second 4 h-digestion.

#### 2.15.2 TMT/iTRAQ labeling

After trypsin digestion, the peptide was desalted using Strata X C18 SPE column (Phenomenex) and vacuum-dried. The peptide was reconstituted in 0.5 M TEAB and processed according to the manufacturer’s protocol for the TMT kit/iTRAQ kit.

#### 2.15.3 HPLC fractionation

The tryptic peptides were fractionated into fractions by high pH reverse-phase HPLC using Thermo Betasil C18 column (5 μm particles, 10 mm ID, 250 mm length).

#### 2.15.4 LC-MS/MS analysis and database search

The tryptic peptides were dissolved in 0.1% formic acid (solvent A), directly loaded onto a home-made reversed-phase analytical column. The gradient was comprised of an increase from 6% to 23% solvent B (0.1% formic acid in 98% acetonitrile) over 26 min, 23% to 35% in 8 min and climbing to 80% in 3 min then holding at 80% for the last 3 min, all at a constant flow rate of 400 nL/min on an EASY-nLC 1000 ultra performance liquid chromatography (UPLC) system. The peptides were then subjected to NSI source followed by tandem mass spectrometry (MS/MS) in Q ExactiveTM Plus coupled online to the UPLC. The resulting MS/MS data were processed using Maxquant search engine (v.1.5.2.8).

#### 2.15.5 Bioinformatic methods

##### 2.15.5.1 Annotation methods

Gene Ontology (GO) annotation proteome was derived from the UniProt-GOA database (http://www.ebi.ac.uk/GOA/). Identified proteins domain functional description was annotated by InterProScan (v.5.14-53.0 http://www.ebi.ac.uk/interpro/). The Kyoto Encyclopedia of Genes and Genomes (KEGG) database was used to annotate the protein pathway. First, using the KEGG online service tool KAAS (v.2.0, http://www.genome.jp/kaas-bin/kaas_main) annotates proteins. Then the annotation results are mapped on the KEGG pathway database using KEGG online service tools KEGG mapper (V2.5, http://www.kegg.jp/kegg/mapper.html).

##### 2.15.5.2 Functional enrichment

Proteins were classified by GO annotation into three categories: biological process, cellular compartment, and molecular function. For each category, a two-tailed Fisher’s exact test was used to test the enrichment of the differentially expressed protein against all identified proteins. GO with a corrected p-value < 0.05 is considered significant. KEGG database was used to identify enriched pathways by a two-tailed Fisher’s exact test to test the enrichment of the differentially expressed protein against all identified proteins. The pathway with a corrected p-value < 0.05 was considered significant. These pathways were classified into hierarchical categories according to the KEGG website. For each category protein, InterPro database was researched and a two-tailed Fisher’s exact test was used to test the enrichment of the differentially expressed protein against all identified proteins. Protein domains with a corrected p-value < 0.05 were considered significant.

##### 2.15.5.3 Enrichment-based clustering

For further hierarchical clustering based on differentially expressed protein functional classification, we collated all the categories obtained after enrichment along with their P values, and then filtered for those categories that were at least enriched in one of the clusters with P value <0.05. This filtered P value matrix was transformed by the function x = −log10 (P value). Then these x values were z-transformed for each functional category. These z scores were then clustered by one-way hierarchical clustering in Genesis. The cluster membership was visualized by a heat map using the “heatmap.2” function from the “gplots” R-package.

### 2.16 Statistical analysis

Statistical analysis and graph presentation were performed using SPSS v.23.0 (IBM Corp., Armonk, NY) and GraphPad Prism 9 software (GraphPad, San Diego, CA), respectively. Continuous data were presented as the mean ± SD and analyzed using Student’s t-tests, analysis of variance (ANOVA), or Mann-Whitney *U* test. Categorical data were compared by Pearson χ2 test or the Fisher exact test. The Kaplan–Meier method and Cox regression were used for univariate and multivariate survival analysis. A two-sided p < 0.05 was considered statistical significance.

## 3 Results

### 3.1 Detection of UCA1 levels in pancreatic tissues by in situ hybridization

**Figure 1:**
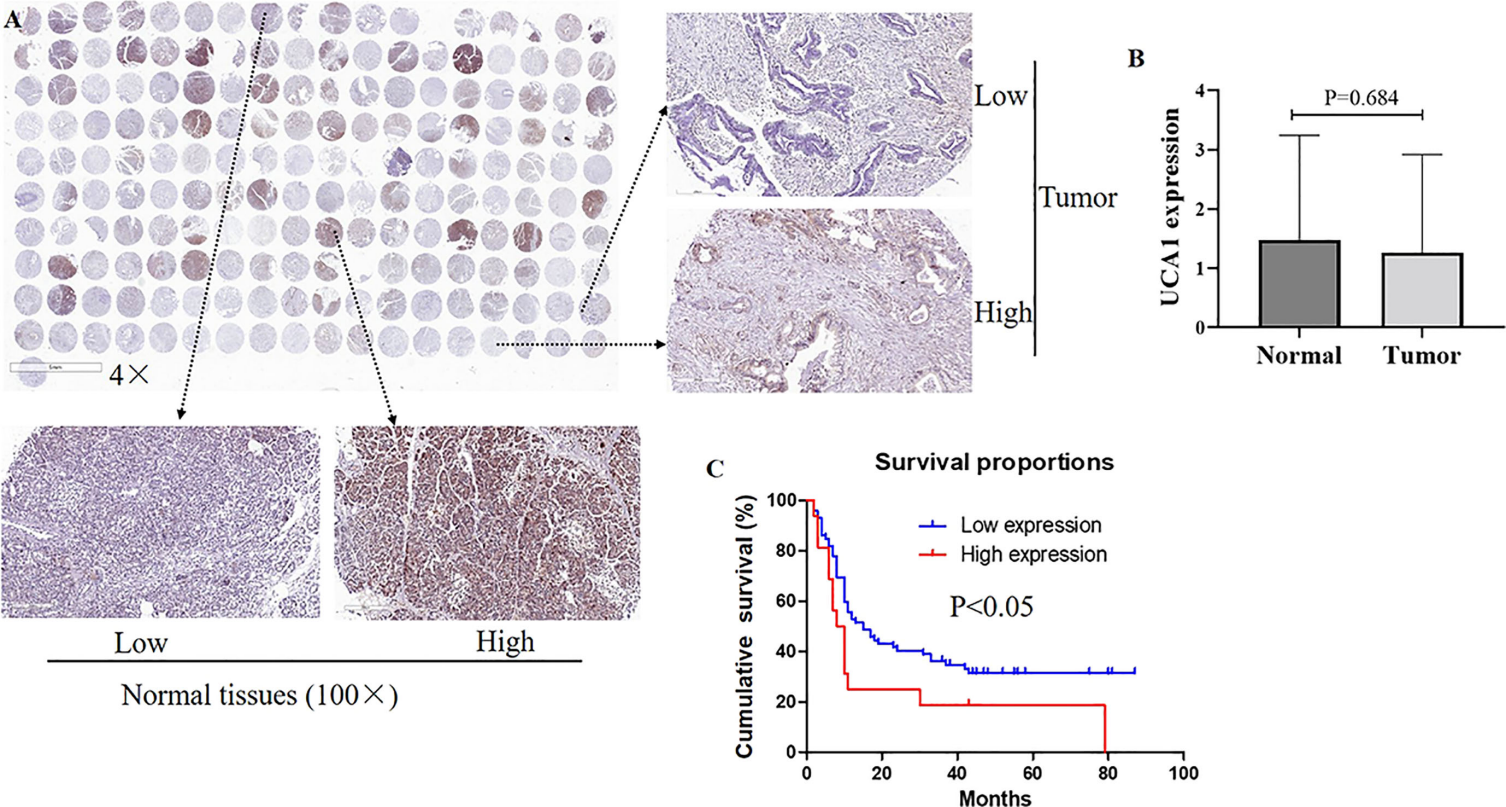
The expression of UCA1 in pancreatic cancer. **(A, B)** Expression levels of UCA1 were detected by ISH [tumor tissues (n=94), normal tissues (n=73)]. Data were analyzed using Mann-Whitney *U* test. **(C)** The survival curve is displayed. Except for six cases without valid follow-up information, 88 cases with pancreatic cancer underwent a further survival analysis.

Pancreatic cancer tissues (n=94) and adjacent normal tissues (n=73) were detected. ISH staining revealed that UCA1 levels between cancer tissues and tumor-adjacent tissues did not show significant differences (p=0.684, **Figure 1**). No correlation was observed between UCA1 levels and clinicopathological parameters, including age, gender, tumor location, differential degree, T stage, N stage, and TNM stage (**Additional File 2**).

Except for six cases without valid follow-up information, 88 cases with pancreatic cancer underwent a further survival analysis. The TNM staging, differential degree, and UCA1 levels were independent prognostic factors (p<0.05). Among these, high expression of UCA1 meant an unfavorable prognosis (p=0.023, hazard ratio=2.172, 95% confidence interval: 1.155–4.068). (**Additional Files 3, 4**).

### 3.2 Downregulation of UCA1 inhibited cell proliferation and induced apoptosis

**Figure 2:**
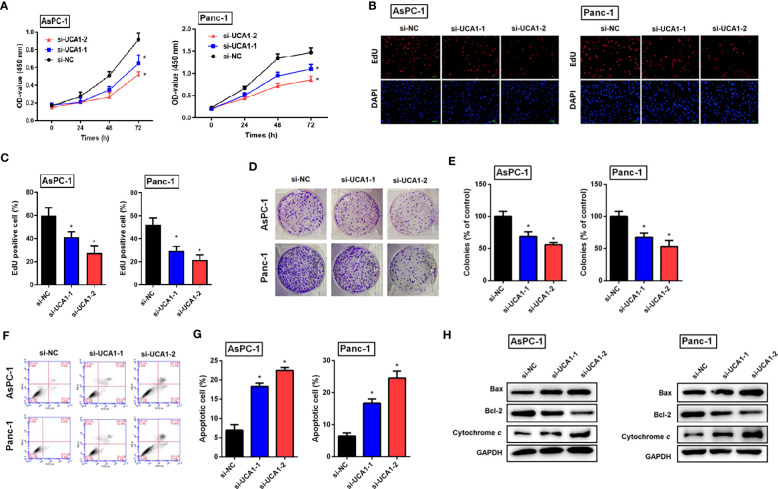
The role of si-UCA1 in cell proliferation and apoptosis in pancreatic cancer. **(A)** Pancreatic cancer cells were transfected with si-UCA1 (n=3) and controls (n=3). Cell proliferation was detected by the CCK8 assay. **(B, C)** EdU experiments indicated the suppression of cell proliferation after UCA1 downregulation. **(D, E)** The colony formation assay indicated the attenuation of cell cloning after UCA1 downregulation. **(F, G)** Cell apoptosis was detected by flow cytometry that indicated an increase in the apoptotic rate after UCA1 downregulation. **(H)** Expression levels of molecules related to apoptosis were detected by western blot. Data were analyzed using Student’s *t*-tests except that presented in panel A, which were analyzed using two-way ANOVA. *P<0.05.

Cell proliferation and viability were evaluated by a CCK-8 assay, an EdU experiment, and a colony formation assay, respectively. All three experiments confirmed that downregulation of UCA1 in pancreatic cancer cells by siRNA suppressed cell proliferation and reduced viability (**Figures 2A–E**).

Cell apoptotic rate was detected by flow cytometry, which indicated that downregulation of UCA1 by siRNA promoted cell apoptosis. Furthermore, downregulation of UCA1 by siRNA inhibited the expression of Bcl-2 (an antiapoptotic molecule) and induced the expression of Bax and cytochrome C (pro-apoptotic molecules) (**Figures 2F–H**).

### 3.3 Downregulation of UCA1 suppressed cell metastasis and EMT

**Figure 3:**
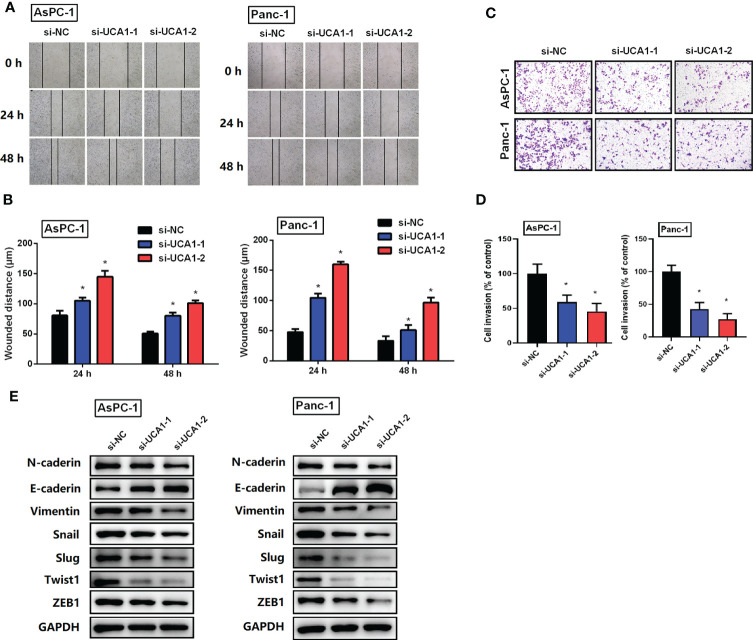
The role of si-UCA1 on cell metastasis. **(A, B)** Cell migration was detected by wound-healing assay, which indicated an inhibition effect of si-UCA1. **(C, D)** Cell invasion detected by the transwell assay was inhibited by si-UCA1. **(E)** The EMT markers were detected by western blot. All the experiments were performed three times. Data were analyzed using Student’s *t*-tests. *P<0.05.

The effects of UCA1 on the migration and invasion of pancreatic cancer cells were examined using a wound healing assay and a transwell assay. Compared with the control group, si-UCA1 inhibited cell migration and invasion, and the wound distance was significantly larger and the number of invaded cells was decreased in si-UCA1 group (**Figures 3A–D**).

Furthermore, considering the vital role of EMT in cancer metastasis, we detected expression levels of EMT markers. We showed that si-UCA1 attenuated the expression levels of N-caderin、Vimentin、Snail、Slug、Twist1 and ZEB1 with the increase of E-caderin (**Figure 3E**).

### 3.4 Downregulation of UCA1 inhibited the growth of xenograft tumors in vivo

**Figure 4:**
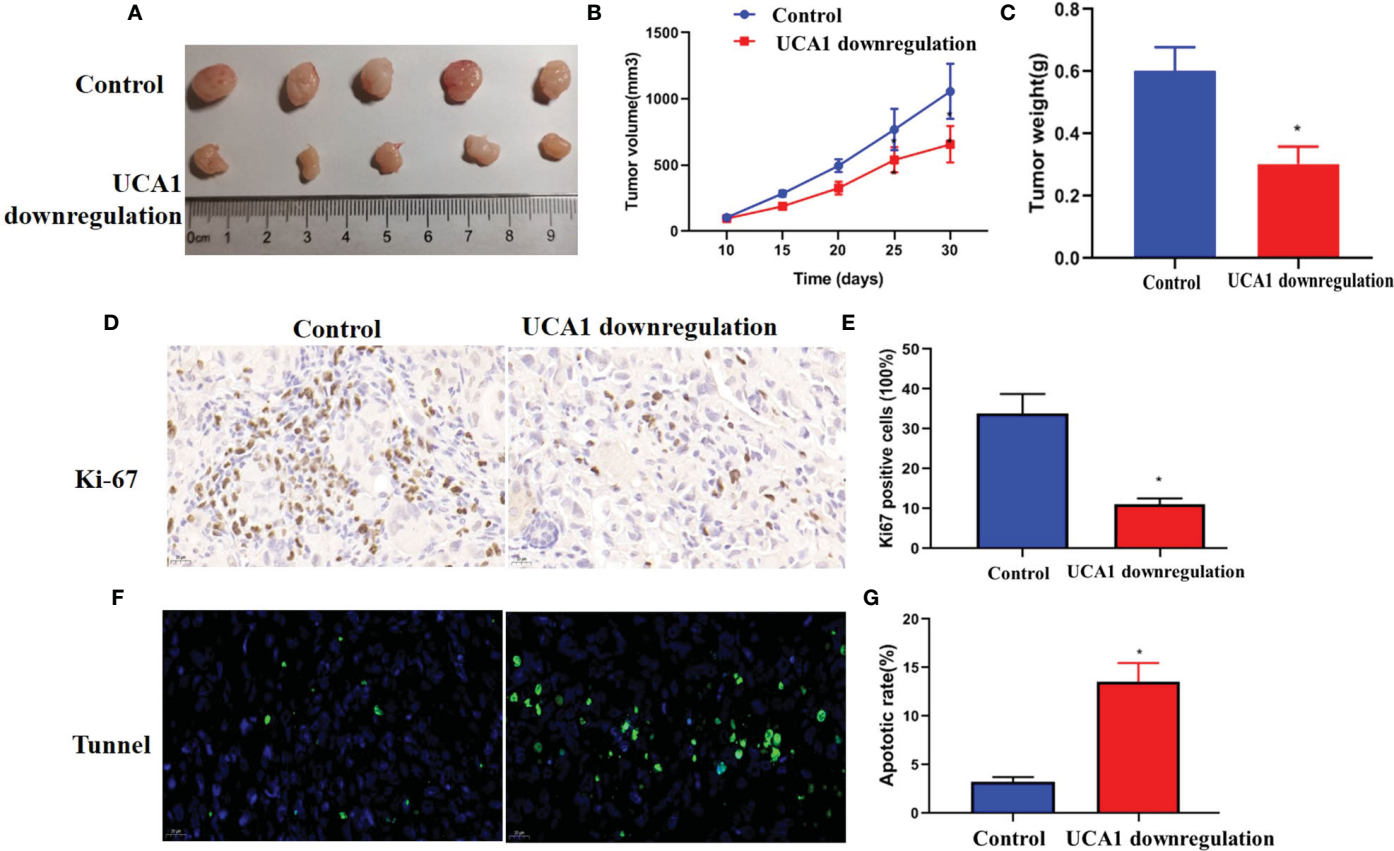
*Iv vivo* experiment. Panc-1 cells with downregulation of UCA1 or control cells were transplanted into five mice, respectively, and tumor growth was monitored. **(A)** The tumor size. **(B)**The growth of the tumors (P<0.05). **(C)** The weight of the tumor samples. **(D, E)** Ki-67 levels in tumors were detected by IHC. **(F, G)** Tumor apoptosis was detected by Tunnel assay. Data were analyzed using Student’s *t*-tests except that presented in panel B, which were analyzed using two-way ANOVA.

*In vivo* models were established to determine the role of UCA1 in tumor growth in mice. Panc-1 cells with UCA1 downregulation or control cells were transplanted into mice, and tumor growth was monitored. The tumor growth, size and weight were significantly decreased in the mice with downregulation of UCA1 than that in control group (**Figures 4A–C**). Ki-67 levels and apoptotic rate in the tumor samples were decreased and increased, respectively (**Figures 4D–G**).

### 3.5 Proteomic analysis

**Figure 5:**
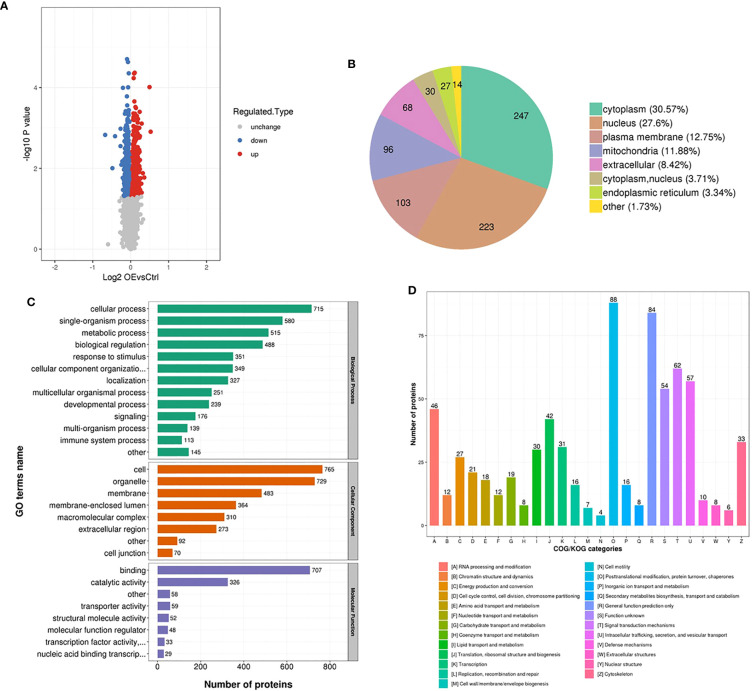
Proteomic analysis. Total proteins were collected from the transfected AsPC-1 cells with the upregulation of UCA1 levels and control cells. Proteomic analysis based on bioinformatics database was performed. **(A)** Number of the dysregulated proteins. **(B)** The locations of dysregulated proteins in cell. **(C)** Function annotation of dysregulated proteins with Gene Ontology. **(D)** Function classification of dysregulated proteins with Clusters of Orthologous Groups of proteins.

Total proteins were collected from the transfected AsPC-1 cells with the upregulation of UCA1 levels and control cells. Proteomic analysis was performed. 5469 proteins were identified, of which 4837 proteins were quantified. A quantitative ratio of the group with upregualted UCA1 levels versus the control group more than one was considered to upregulation, while the ratio of less than one was considered to downregulation. 460 proteins and 348 proteins were significantly upregulated and downregulated, respectively (**Figure 5A**). The annotation and quantification information are listed in **Additional File 5**. Of that, only 7 proteins with a ratio greater than 1.2 and 5 proteins with a ratio less than 1/1.2 were identified (**Additional File 6**).

The differentially expressed proteins were mainly located in cytoplasm and nucleus (**Figure 5B**), functionally annotated with Gene Ontology (GO) in cellular and single-organism process, binding and catalytic activity (**Figure 5C**), functionally classified with Clusters of Orthologous Groups of proteins (COG) in posttranslational modification, protein turnover, chaperones (**Figure 5D**). Given the expression levels, P values, potential functions, and interaction between UCA1, miRNA, and protein, BRCC3 was selected as a candidate to investigate.

### 3.6 Verification of the regulatory net of UCA1/miR-582-5p/BRCC3

**Figure 6:**
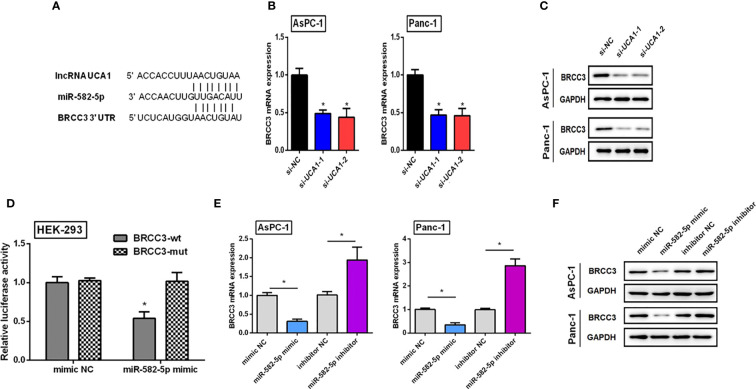
Verifying the regulatory net of UCA1/miR-582-5p/BRCC3. **(A)** Bioinformatics analysis indicated that both UCA1 and BRCC3 had the same miRNA response element. **(B, C)** The expression of BRCC3 mRNA and protein was detected by qRT-PCR and western blot after downregulation of UCA1, respectively. **(D)** Dual luciferase assay indicated that BRCC3 was a direct target of miR-582-5p. **(E, F)** Expression levels of BRCC3 mRNA and protein by qRT-PCR and western blot after transfection of miR-582-5p mimics or inhibitors. All the experiments were performed three times. Data were analyzed using Student’s *t*-tests *P<0.05.

Bioinformatic analysis was performed and a regulatory net of UCA1/miR-582-5p/BRCC3 was identified (**Figure 6A**).

#### 3.6.1 The influence of UCA1 on the expression of BRCC3

Compared with the control group, si-UCA1 significantly decreased BRCC3 expression levels (**Figures 6B, C**).

#### 3.6.2 miR-582-5p directly binding to the 3’-UTR of BRCC3

We generated luciferase reporter constructs containing either the wild-type or mutated binding sequences in BRCC3 downstream of the firefly luciferase gene. HEK-293 cells were cotransfected with reporter vectors and miR-582-5p mimics or control, and luciferase activities were evaluated. Luciferase activities were significantly decreased after cotransfection of miR-582-5p mimics with vectors containing wild-type binding site sequences of BRCC3 compared with cells cotransfected with mimics and vectors containing the mutated binding site sequence. Luciferase activities were also reduced compared with cells transfected with vectors containing wild-type binding sequences and control, as well as cells with mutant binding sequence vectors and control (**Figure 6D**). These results indicated that BRCC3 was a direct target of miR-582-5p.

#### 3.6.3 The influence of miR-582-5p on BRCC3 expression

Pancreatic cancer cells were treated with miR-582-5p mimics, inhibitors, and controls. Compared with controls, mimics decreased BRCC3 expression levels, and inhibitors promoted BRCC3 expression (**Figures 6E, F**).

### 3.7 Rescue experiments

To further confirm the regulatory net of UCA1/miR-582-5p/BRCC3, we performed rescue experiments through bidirectional regulation of gene expression.

#### 3.7.1 Simultaneously inhibited the expression levels of UCA1 and miR-582-5p in pancreatic cancer cells, and then observed cell proliferation, apoptosis, and metastasis

**Figure 7:**
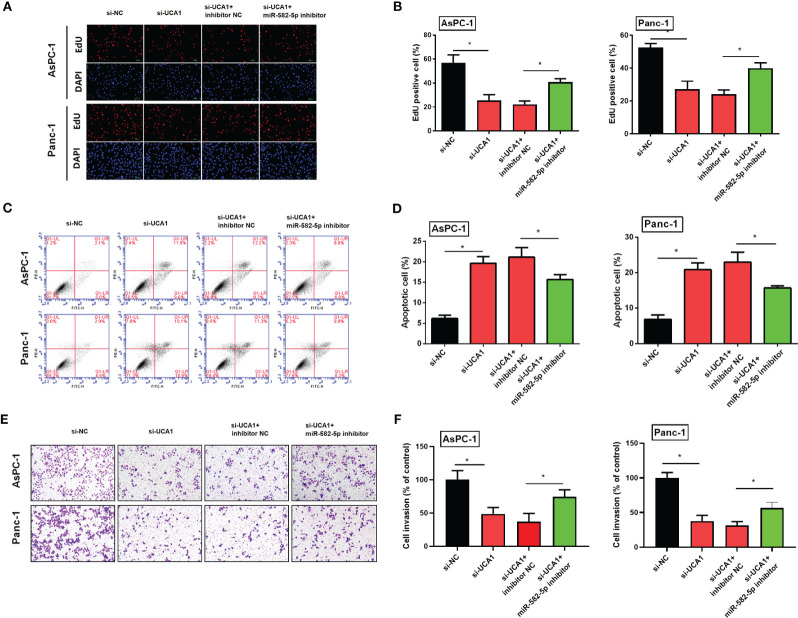
Simultaneously inhibited the expression levels of UCA1 and miR-582-5p in pancreatic cancer cells, and then observed the cell proliferation, apoptosis, and metastasis. **(A, B)** Cell proliferation was detected by EdU experiment. **(C, D)** Cell apoptosis was detected by flow cytometry. **(E, F)** Cell invasion was detected by transwell assay. All the experiments were performed three times. Data were analyzed using two-way ANOVA. *P<0.05.

Compared with pancreatic cancer cells with si-UCA1 alone, cells were cotransfected with si-UCA1 and miR-582-5p inhibitors showed increased proliferation and metastasis, and decreased apoptosis (**Figure 7**). These results meant that the inhibited effects of si-UCA1 on tumor progression were partly attenuated after cotransfection of si-UCA1 and miR-582-5p inhibitors.

#### 3.7.2 Simultaneously inhibited the expression of UCA1 and enhanced the expression of BRCC3 in pancreatic cancer cells, and then observed cell proliferation, apoptosis, and metastasis

**Figure 8:**
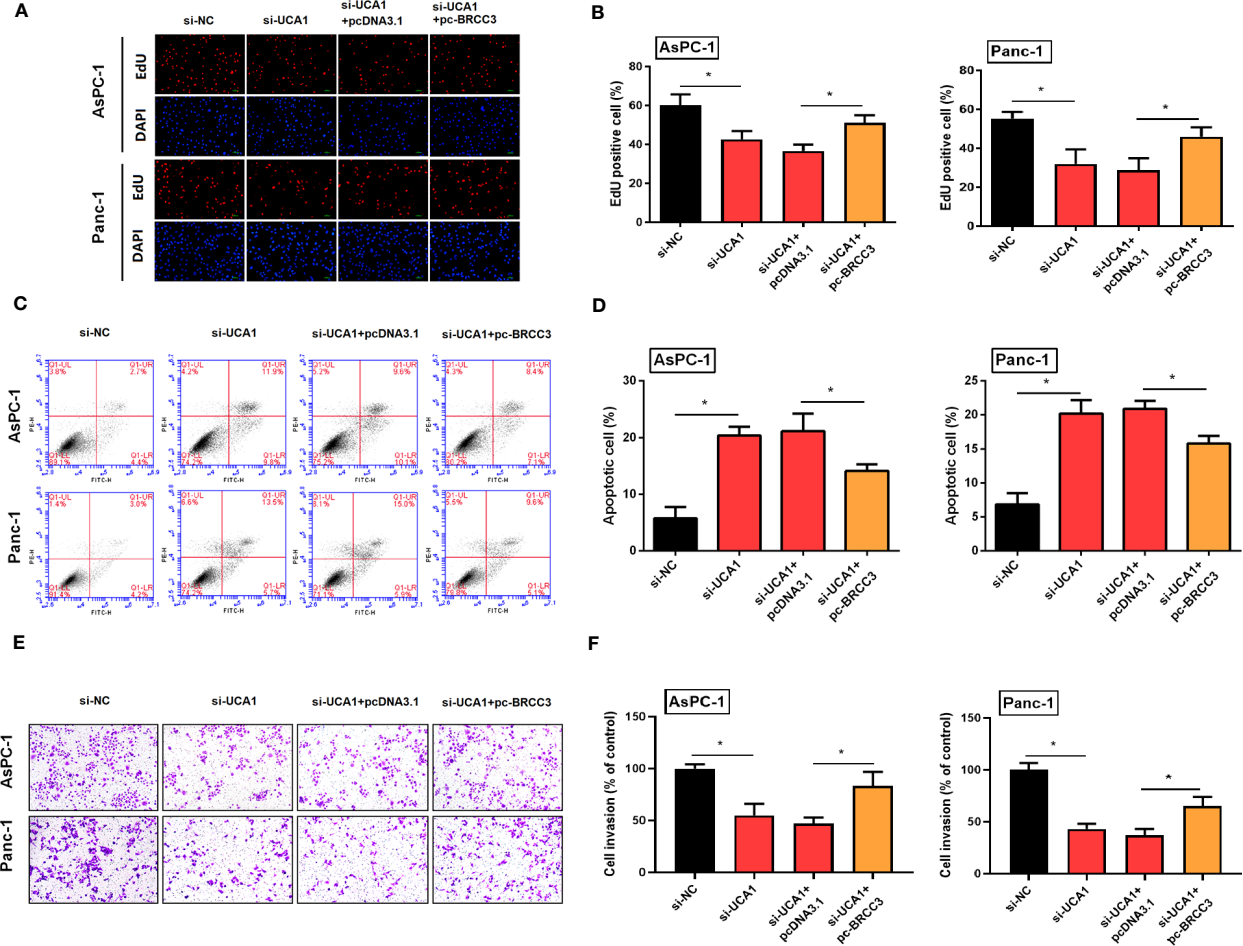
Simultaneously inhibited the expression of UCA1 and enhanced the expression of BRCC3 in pancreatic cancer cells, and then observed the cell proliferation, apoptosis, and metastasis. **(A, B)** Cell proliferation was detected by EdU experiment. **(C, D)** Cell apoptosis was detected by flow cytometry. **(E, F)** Cell invasion was detected by transwell assay. All the experiments were performed three times. Data were analyzed using two-way ANOVA. *P<0.05.

Compared with pancreatic cancer cells with UCA1 inhibitors only, cells were co-transfected with si-UCA1 and pc-BRCC3 (a plasmid for upregulation of BRCC3) showed increased cell proliferation and metastasis, and decreased in apoptosis (**Figure 8**). These results meant that the inhibited effects of si-UCA1 on tumor progression were partly attenuated after cotransfection of si-UCA1 and pc-BRCC3.

## 4 Discussion

Pancreatic cancer is difficult to diagnose in the early stage with high degree of malignancy and a poor prognosis. Understanding the mechanism of its occurrence and development is beneficial to overcome this fatal disease. Our study investigated the regulatory role of UCA1 in pancreatic cancer. We showed that although there were no significant differences in between pancreatic cancer tissues and the para-cancerous tissues, UCA1 expression levels were negatively correlated with the prognosis of pancreatic cancer patients. Functionally, UCA1 carried out an oncogenic role by regulating the miR-582-5p/BRCC3 signal to promote tumor progression.

The expression level of UCA1 in cancer tissues was still controversial. Most literatures had reported that the expression level of UCA1 in the majority digestive tract tumors such as gastric cancer, hepatocellular carcinoma and colon cancer was higher than that in adjacent tissues (7). However, UCA1 acted as a tumor suppressor gene in the esophageal cancer, the expression level is lower in the cancer tissues than that in the adjacent tissues (16). The expression level of UCA in pancreatic cancer was also reported. Bioinformatic analysis with small sample validation suggested the higher expression level of UCA1 in cancer tissues than in adjacent tissues (11, 17). A large sample study also confirmed the higher expression of UCA1 in the pancreatic cancer tissues by qRT-PCR (18). However, our study cannot observe the difference in UCA1 expression between cancer and adjacent tissues. This inconsistent results with previous studies might be explained by the sample type and size, detection method, and the scoring criteria.

For the prognostic value of UCA1 in cancer tissues, the conclusions of current studies were relatively consistent. A meta-analysis that included a total of 16 studies with 1504 patients that contained 6 studies on colorectal cancer, 3 studies on gastric cancer, 3 studies on pancreatic cancer, and 2 studies on hepatocellular carcinoma and esophageal cancer indicated that patients with a high expression of UCA1 in cancer tissues had a poor prognosis. The overall survival and disease-free survival of these patients were inferior to those of patients with low expression of UCA1 (19).

Some studies have reported the regulatory role of UCA1 in pancreatic cancer, which showed that UCA1 promoted cell proliferation and angiogenesis by regulating miR-590-3p/Kras (11), miR-107/ITAG2 (20), and miR-96/FOXO3 (12). Even so, little was known about the function and regulatory mechanisms of UCA1 in pancreatic cancer. Our study reported a novel signaling pathway that UCA1 promoted tumor progression by regulating miR-582-5p/BRCC3. To my knowledge, this was the first report on the regulatory roles of UCA1 and miR-582-5p on BRCC3.

BRCC3 has been less investigated in tumors. A study indicated that the expression of BRCC3 was increased in bladder cancer, which was associated with a poor prognosis (21). BRCC3 led to the resistance of glioma cells to temozolomide (22). Downregulation of BRCC3 inhibited cell proliferation and metastasis by inhibiting EMT of cervical cancer cells (23). Our study was the first to report the function of BRCC3 in pancreatic cancer, which found that downregulation of BRCC3 inhibited the cell proliferation, metastasis and induced apoptosis. The mechanism of BRCC3 in promoting tumor progression was unclear. Tao et al. (21) reported that BRCC3 enhanced cell proliferation and migration by activating TRAF2/NF-κB pathway. Boudreau et al. (24) demonstrated that downregulation of BRCC3 inhibited cell proliferation via attenuating p-ERK expression. Hu et al. (25) reported that the TMPO-AS1/miR-126-5p/BRCC3 axis promoted gastric cancer progression by activating the PI3K/Akt/mTOR pathway. Besides, BRCC3 could activate NLRP3 inflammasome-dependent pyroptosis (26), which could then promote tumor progression.

In conclusion, UCA1 acted as an oncogene in pancreatic cancer by partly regulating miR-582-5p/BRCC3, which promoted cell proliferation, migration, and inhibited apoptosis. The high expression of UCA1 in cancer tissues indicated a poor prognosis. UCA1 could be a potential therapeutic target and prognostic marker for pancreatic cancer.

## Data availability statement

The datasets presented in this study can be found in online repositories. The names of the repository/repositories and accession number(s) can be found in the article/**Supplementary Material**.

## Ethics statement

The studies involving human participants were reviewed and approved by the Medical Ethics Committee of Qilu Hospital of Shandong University. The patients/participants provided their written informed consent to participate in this study. The animal study was reviewed and approved by the Institutional Animal Care and Use Committee of Qilu Hospital of Shandong University.

## Author contributions

JX proposed and designed the study. XH, JW, and JX carried out the experiments. XH and JX collected and analyzed the data. XH and JW wrote the draft. JX revised the manuscript. All authors contributed to the article and approved the submitted version.
