# Supplementary material for: UCA1 executes an oncogenic role in pancreatic cancer by regulating miR-582-5p/BRCC3
Source: Front Oncol. 2023 Jul 25;13:1133200. doi: 10.3389/fonc.2023.1133200 (PMC10411552; doi:10.3389/fonc.2023.1133200)
Supplement: Supplementary file 2 [file Table_2.docx]

**Additional file 2 The correlation between UCA1 levels and clinicopathological parameters**

| **Variables** | **Tissue UCA1 expression** | | **P value** |
| --- | --- | --- | --- |
|  | **Low group**  **(N=72)** | **High group**  **(N=16)** |  |
| **Gender** |  |  | 0.791 |
| Male | 47 | 11 |  |
| Female | 25 | 5 |  |
| **Age（years old）** |  |  | 0.918 |
| ＜65 | 44 | 10 |  |
| ≥65 | 28 | 6 |  |
| **Vessel invasion** |  |  | 0.168 |
| Yes | 27 | 9 |  |
| No | 45 | 7 |  |
| **Differential degree** |  |  | 1.000 |
| High /Moderate | 52 | 12 |  |
| Low | 20 | 4 |  |
| **Tumor staging** |  |  | 0.176 |
| T1/T2 | 54 | 15 |  |
| T3/T4 | 18 | 1 |  |
| **Lymph node staging** |  |  | 0.642 |
| N0 | 45 | 9 |  |
| N1 | 27 | 7 |  |
| **TNM staging** |  |  | 0.719 |
| Ⅰ | 28 | 7 |  |
| Ⅱ/Ⅲ/Ⅳ | 44 | 9 |  |
