# Supplementary material for: UCA1 executes an oncogenic role in pancreatic cancer by regulating miR-582-5p/BRCC3
Source: Front Oncol. 2023 Jul 25;13:1133200. doi: 10.3389/fonc.2023.1133200 (PMC10411552; doi:10.3389/fonc.2023.1133200)
Supplement: Supplementary file 3 [file Table_3.docx]

**Additional file 3 Univariate survival analysis**

| **Variables** | **Overall survival time**  **(Median±SE，months)** | **95% Confidence Interval（CI）** | **1-year survival (%)** | **P value** |
| --- | --- | --- | --- | --- |
| **Sex** |  |  |  | 0.226 |
| Male | 10.0±1.3 | 7.5-12.5 | 43.1 |  |
| Female | 19.0±8.9 | 1.6-36.4 | 56.7 |  |
| **Age（years old）** |  |  |  | 0.372 |
| ＜65 | 13.0±2.9 | 7.4-18.6 | 51.9 |  |
| ≥65 | 10.0±1.4 | 7.2-12.8 | 41.2 |  |
| **Vessel invasion** |  |  |  | 0.260 |
| Yes | 10.0±0.9 | 8.2-11.8 | 41.7 |  |
| No | 13.0±3.6 | 5.9-20.1 | 51.9 |  |
| **Differential degree** |  |  |  | 0.002 |
| High/ Moderate | 15.0±5.8 | 3.7-26.3 | 54.7 |  |
| Low | 7.0±1.2 | 4.6-9.4 | 58.3 |  |
| **Tumor staging** |  |  |  | 0.818 |
| T1/T2 | 11.0±1.2 | 8.7-13.3 | 44.9 |  |
| T3/T4 | 19±9.4 | 0.5-37.5 | 57.9 |  |
| **Lymph node staging** |  |  |  | 0.003 |
| N0 | 30.0±10.5 | 9.4-50.6 | 59.3 |  |
| N1 | 10.0±1.2 | 7.6-12.4 | 29.4 |  |
| **TNM staging** |  |  |  | 0.002 |
| Ⅰ | 42.0±20.0 | 2.8-81.2 | 65.7 |  |
| Ⅱ/Ⅲ/Ⅳ | 10.0±1.0 | 8.1-11.9 | 35.8 |  |
| **UCA1 expression** |  |  |  | 0.049 |
| Low | 15.0±3.7 | 7.7-22.3 | 52.8 |  |
| High | 8.0±1.5 | 5.1-10.9 | 25.9 |  |
