# Supplementary material for: UCA1 executes an oncogenic role in pancreatic cancer by regulating miR-582-5p/BRCC3
Source: Front Oncol. 2023 Jul 25;13:1133200. doi: 10.3389/fonc.2023.1133200 (PMC10411552; doi:10.3389/fonc.2023.1133200)
Supplement: Supplementary file 4 [file Table_4.docx]

**Additional file 4** **Multivariate survival analysis**

| **Variables** | **Hazard ration (HR)** | **95%Confidence interval (CI)** | | **P value** |
| --- | --- | --- | --- | --- |
|  |  | **Lower** | **Upper** |  |
| **TNM staging (Ⅱ/Ⅲ/Ⅳ)** | 2.130 | 1.034 | 4.390 | 0.044 |
| **UCA1 expression (High)** | 2.172 | 1.155 | 4.086 | 0.023 |
| **Lymph node staging (N1)** | 1.780 | 0.914 | 3.466 | 0.082 |
| **Differential degree (Low)** | 2.838 | 1.615 | 4.987 | 0.001 |
